# What factors are most influential in increasing cervical cancer screening attendance? An online study of UK-based women

**DOI:** 10.1080/21642850.2020.1798239

**Published:** 2020-08-07

**Authors:** Sarah Wilding, Sarah Wighton, Daisy Halligan, Robert West, Mark Conner, Daryl B. O’Connor

**Affiliations:** a School of Psychology, University of Leeds, Leeds, UK; b NHS England and NHS Improvement (North East & Yorkshire), Leeds, UK; c Leeds Institute of Health Sciences, University of Leeds, Leeds, UK

**Keywords:** Cervical cancer screening, decision making, decision making in cervical cancer screening, barrier, facilitator

## Abstract

**Objective**: Cervical cancer is the fourth most commonly occurring cancer in women worldwide. The UK has one of the highest cervical screening rates in Europe, yet attendance has been decreasing. This study aimed to identify barriers and facilitators to screening attendance and assess the perceived importance of these factors.

**Methods**: 194 women living in the UK were recruited via an online research recruitment website to an online survey. Most participants (*N* = 128, 66.0%) were currently up-to-date with cervical screening, 66 participants (34.0%) had never been screened, or were overdue for screening. Participants identified barriers and facilitators to cervical screening attendance via free-text responses and were also asked to rate a list of factors as most to least influential over decision making. Results were analysed using thematic content analysis and ratings analysed using multivariable analyses.

**Results**: The most commonly reported barriers were: Pain/discomfort; Embarrassment; and Time. These were also rated as most influential for decision making. The most commonly reported facilitators were: Ease of making appointments; Peace of mind; and Fear of cancer/preventing serious illness. While importance rating of barriers did not differ by previous screening behaviour, ratings of some facilitators significantly differed. Up-to-date women rated believing screening is potentially life-saving and part of personal responsibility as significantly more important than overdue/never screened women.

**Conclusion**: This study confirmed that factors which encourage screening are key to the decision of whether to attend screening. Women suggested several improvements that might make attending easier and improve uptake, including flexibility of screening locations to fit around work hours and childcare arrangements. Psychological facilitators included the peace of mind that screening brings and the belief that cervical cancer screening is potentially life-saving. Public health interventions should target factors which facilitate screening and how these interplay with barriers in order to improve uptake.

## Introduction

Cervical cancer is the fourth most common cancer to occur in women worldwide (World Health Organisation, 2018). Regular screening is supported as a method to reduce cervical cancer incidence and subsequent mortality (Peirson, Fitzpatrick-Lewis, Ciliska, & Warren, 2013). EU recommendations suggest cervical cancer screening should be offered on a population-level basis in organised screening programmes (European Council, 2003). At present, Sweden, the United Kingdom, and Norway report uptake rates around 70% which are the highest across Europe, however no countries report attendance at or above the 85% rate recommended (Gianino et al., 2018).

The National Health Service (NHS) cervical screening programme in the UK routinely sends invitations, re-invitations and reminders to take part in the screening to eligible women, while appointments are organised with their local general practitioner. In the UK, eligible individuals are invited every three (if aged 25–49 years) or five years (if aged 50–64 years). Cervical screening rates have been decreasing year on year (Douglas, Waller, Duffy, & Wardle, 2016) and current coverage ranges from 72% to 76.4% across the countries of the UK, with the lowest rate reported in England and the highest rate reported in Northern Ireland (Cervical Screening Wales, 2019; HSC Public Health Agency, 2018; Public Health Scotland, 2020). Cervical cancer incidence across the UK is highest in women aged 25–29 years and those living in areas of greatest deprivation (Cancer Research UK, 2015). Despite this, screening uptake is lowest in these groups (Douglas et al., 2016; NHS Digital, 2019).

In order to reduce health inequalities associated with deprivation and age, it is important to understand the underlying reasons behind screening non-attendance. Reviews of barriers to screening attendance have demonstrated a wide range of barriers including lack of knowledge, embarrassment, fear of pain, and logistical factors (Bukowska-Durawa & Luszczynska, 2014; Chorley, Marlow, Forster, Haddrell, & Waller, 2017; Ferdous et al., 2018; Hope, Moss, Redman, & Sherman, 2017; Ramjan, Cotton, Algoso, & Peters, 2016). One systematic review of correlational and experimental studies including at least 50 participants identified 53 barriers listed in at least two of the correlational studies, these were categorised into seven categories (facilities, personal barriers, beliefs, awareness, emotional, social, social support). The majority of studies reviewed were located in the U.S.A. and a quarter of studies reported long distance/transportation as a barrier to screening. Additional barriers included lack of childcare, managing other priorities, and procrastination (Bukowska-Durawa & Luszczynska, 2014). However, no studies provided insight into which barriers were most influential over decision making.

Little attention has been paid to the factors which encourage or facilitate screening attendance (O’Connor, Murphy, Martin, O’Leary, & Sharp, 2014). One qualitative study using face to face and online focus groups to explore what factors encourage 30-year-old Swedish women to engage in cervical cancer screening found a range of factors were considered to facilitate screening in a group of 138 women, including invitations to screening and reminder letters along with providing a more individualised screening process (Blomberg et al., 2010). Focus groups conducted in a group of Irish women prior to the introduction of a national cervical screening programme, questioned the factors which would motivate women to attend and also demonstrated that personal beliefs encouraged participation, including considering the screening to be life-saving (O’Connor et al., 2014).

The UK has one of the highest uptake rates for cervical cancer screening in Europe. By better understanding the factors that encourage eligible individuals from the UK along with the barriers that prevent some women from attending, this will enable us to understand what is currently effective along with what improvements still need to be made, particularly in the context of declining rates in screening over recent years.

The present study aims to add to the existing body of literature in three ways. First, using an anonymous, online survey method targeting a wide range of women in terms of age, socioeconomic deprivation, and past screening behaviour, the study aimed to qualitatively assess the barriers and facilitators in a sample not limited by these key characteristics. The free-text nature of the responses requested also allowed participants to identify the reasons they saw as influential along with providing some explanation of why these were influential. The benefits of using an online survey to capture qualitative data include recruitment of a larger number of women overall, along with individuals who otherwise might be put off from attending face to face methods. This is a particular concern with women aged 25–64 who are of working age and may be limited to take part in research due to competing demands for their time, in particular juggling work along with potential childcare commitments.

Second, there has been little focus on the factors which encourage screening attendance, particularly in UK samples. The most recent studies to focus on this were conducted over 6 years ago and with declining screening rates, it is important to understand the current factors which influence decision making. The present study therefore asked participants to identify both barriers and facilitators to screening attendance. Third and finally, a wide range of influential factors have been identified in previous research, however, many of these are identified by both attenders and non-attenders. It is unclear whether some factors are more influential over decision-making or whether there are key differences in the barriers and facilitators identified by up-to-date vs. non-attenders/women overdue for screening.

## Method

### Participants

Participants were women living in the UK recruited via Prolific (https://prolific.ac/), an online research recruitment website which guarantees that participants are paid a minimum of £5.00 p/h for completing surveys. The database of potential participants has approximately 18,000 women aged 25–64 who currently reside in the UK, 90.5% of these women are White. We screened 500 women with the aim of recruiting approximately 25 women in each of the eight cells (total *N* = 200) defined by socioeconomic status (lower 50% deprivation deciles vs. higher 50% deprivation deciles), age (younger: 25–29 vs. older: 30–65 years) and previous screening behaviour (up-to-date vs. overdue/never screened). This stratification aimed to recruit women with a range of background characteristics to enable comparison in women who, based on background literature, tend to be less likely to attend screening.

194 women completed the main survey (mean age = 33.9; SD = 9.00), 94 women were aged 25–29 (48.4%) and 100 were aged 30–65 (51.6%). All women reported having heard of cervical cancer screening before.

Participants were categorised as up-to-date with screening if they were aged 25–49 and had been screened in the past 3 years, or were aged 50–64 and had been screened in the past 5 years. The majority of participants were up-to-date with screening (*N* = 128, 66%), 66 participants were overdue/never screened (34%; Table 1). Of the overdue/never screened women, 24 (36.4%) were from the younger age group and 42 (63.6%) were from the older age group. Just under 90% of participants (89.2%; *N* = 174) were Caucasian with the remaining 20 participants from non-white ethnicities.

**Table 1.** Total number of participants in each of the pre-defined subgroups.

Previous screening behaviour			IMD^†^ quintile					Total
			Most deprived	2	3	4	Least deprived	
Up-to-date^‡^	Age group	Younger (25–30)	12	14	17	6	4	53
		Older (30–65)	11	10	5	7	12	45
Overdue/Never screened	Age group	Younger (25–30)	3	10	2	2	1	18
		Older (30–65)	3	8	12	3	3	29
Total	Age group	Younger (25–30)	15	24	19	8	5	71
		Older (30–65)	14	18	17	10	15	74
	Total		29	42	36	18	20	145

Notes: ^†^IMD Index of multiple deprivation based on participant postcodes. 145 participants provided postcodes which could be converted into IMD quintiles. ^‡^Participants were classified as up-to-date with screening if they were aged 25–49 and had been screened in the past 3 years, or were aged 50–65 and had been screened in the past 5 years.

### Measures

Participants were asked to complete an online survey about their thoughts on cervical cancer screening. They received £1.00 (approx. $1.33) for taking part in a 10-minute survey (rate of £6.00/h). Participants provided demographic information including age, ethnicity, nationality, postcode, whether they were currently registered with a UK GP, and when they last attended for screening. Postcodes were transformed into index of multiple deprivation quintiles. The measures of IMD deprivation differ slightly across the countries of the UK, but are made up of a composite of factors including income, education and employment within a specific postcode area.145 women provided postcode information. The online survey had two parts, the first was a free-text component asked participants to identify up to five barriers and five facilitators to attending screening and also asked participants to explain why these influenced their decision-making. Participants were asked to list the ‘sorts of things that might put you off going for screening’ with free-text response boxes asking them to list between three and five reasons and to provide an explanation as to why each might put them off attending screening. The second part of the survey provided a list of 10 barriers and 10 facilitators taken from the literature and asked participants to rate these from 10 (most important) to 1 (least important) to decision making. The list of barriers and facilitators are reported in Table 3. The survey was designed to initially allow participants first to identify the factors that influenced their decision making in their own words and to provide as much explanation as to why this influenced their decision. The second part of the survey was designed to test whether specific barriers and facilitators identified through previous research varied in the perceived level of influence over the decision of whether or not to attend screening.

### Ethical approval

Approval was granted by the University of Leeds, School of Psychology Ethics Committee (Ref: PSC-645, Date: 19/03/2019). All participants provided informed consent prior to completing the online survey.

### Analysis summary

A thematic content analysis was conducted (by SW) to identify the barriers and facilitators to screening most commonly reported by participants in response to the free-text questions, following the steps outlined by Elo and Kyngäs (2008). Responses were read to ensure familiarity with the material and its context. Free-text comments were coded by identifying recurring words or units of meaning. The frequency of reporting each of these codes was then calculated. Codes were grouped into categories and reread and compared to check for consistency of meaning based on the context of the comments. The frequency of reporting of these categories was calculated. This process was conducted initially on the overall sample of women, and repeated limited to the women who were overdue for screening/had never been screened (Supplementary Tables 3–4).

Coding and categorical decisions were checked for a random 5% of the comments (by DH), discrepancies or disagreements were discussed, and any necessary adjustments were made. Higher-order categories and codes are presented in Supplementary Table 1, illustrated by quotes. In order to preserve context, comments are presented in full. Some comments fit into more than one code and category. Also reported are the frequencies of reporting each of the categories, these frequencies were also compared across the three subgroups of age, deprivation, and previous screening behaviour using Chi-squared analyses. The 10 most commonly reported barriers and facilitators identified by the free-text responses are reported below, each illustrated by a single quote.

The ratings of the 10 facilitators and 10 barriers were analysed using descriptive statistics, ratings were then compared by age, deprivation, and previous screening behaviour using multivariate analysis of variance. Quantitative analyses were conducted using SPSS v.23.

## Results

### Free-text responses

#### Barriers

Participants reported 41 barriers in total. Presented below are the 10 most commonly reported categories of barriers reported in response to the free-text question (number and % of women reporting) along with a quote from the free-text responses illustrate each of these. The full list of quotes is available in Supplementary Table 1.

1. Pain/discomfort (*n* = 131; 67.2%), including previous experience of pain, or the fear of potential pain; ‘Wanting to avoid any pain or discomfort.’
2. Embarrassment (*n* = 113; 57.9%), including anxiety and embarrassment about how their body looks; ‘Find the whole process embarrassing.’
3. Time (*n* = 95; 48.7%), this included lack of time, having other commitments, being too busy, and finding making the appointments inconvenient; ‘It is hard to find time during my regular working hours to schedule a screening, as I work 7 days a week.’
4. Appointment availability (*n* = 68; 34.9%), including difficulty making an appointment and requiring childcare during the appointment; ‘My GP surgery operated between 8 and 5 weekdays only, it is very difficult to get an appointment when I work full time. It is made even more difficult as the nurses are not available every day, only on certain days during the week.’
5. Fear of results (*n* = 59; 30.3%); ‘Fear of finding out something is actually wrong.’
6. Body issues (*n* = 35; 17.9%), including worry about how their body looks and issues with irregular periods making screening difficult; ‘Not wanting to have to take clothes off in front of people (even if they are medical professionals who’ve “seen it all before”) due to body issues.’
7. Being at low perceived risk (*n* = 30; 15.4%), including due to a lack of sexual activity, or not perceiving that they do not need screening; ‘I almost certainly do not have HPV due to my limited sexual contact. My husband and I have only ever had sexual contact with each other.’
8. Being nervous or unsure of the screening process or what is involved (*n* = 20; 10.3%); ‘Scary to have an invasive process when it is unknown what it will be like’
9. Previous experience (*n* = 18; 9.2%), including having had a negative experience of screening itself, or other experiences (including sexual assault) which have influenced their subsequent experiences; ‘very first screening was an uncomfortable experience that left me in discomfort for several days afterward.’
10. GP or other staff-related factors (*n* = 16; 8.2%), which included worry about potentially having a male healthcare professional complete the screening, and not trusting their GP; ‘When I have attended other appointments the nurse or practitioner has not been warm or friendly to encounter. This always puts me going to the doctor regardless of the issue.’

Comments which did not fit into any of these broad categories included having experienced previous sexual assault (*n* = 3; 1.5%), disliking being touched (*n* = 2; 1.0%), issues with childbirth (*n* = 4; 2.1%) and having heard horror stories in relation to screening (*n* = 4; 2.1%).

#### Subgroup comparisons

Chi square subgroup analyses were conducted to compare the frequency of barrier reporting by age, deprivation and previous screening behaviour. Pain/fear of pain was the most commonly reported barrier in all three subgroups, followed by embarrassment, and time. Significant subgroup differences were found for two of the barriers. Older participants were significantly more likely to report appointment availability as a barrier (41.0%) compared to younger participants (28.4%), Χ^2^ (1, *N* = 194) = 3.39, *p* = .04. Older participants were also significantly more likely to report previous experience as a barrier (13.0%) compared to younger participants (5.3%), Χ^2^ (1, *N* = 194) = 3.48, *p* = .05.

#### Facilitators

A total of 46 facilitators were reported. The 10 most commonly reported categories of facilitators reported are listed below along with a quote from the free-text responses to illustrate each facilitator (number and % of women reporting). The full list of quotes representing each facilitator is available in Supplementary Table 2:

1. Ease of making appointments (*n* = 71; 36.4%), including being able to make appointments at alternative locations; ‘Being able to attend a clinic that does not interfere with my other commitments.’
2. Peace of mind (*n* = 48; 24.6%), including knowing that they were safe and wanting the results; ‘Just to be on the safe side.’
3. Fear of cancer or to prevent serious illness (*n* = 48; 24.6%), which included being conscious of catching the disease early and being aware that screening is potentially lifesaving; ‘It’s a free, potentially lifesaving, simple check-up. Why would I not have it done?’
4. Perceived risk of cancer (*n* = 38; 19.5%), including wanting to minimise the risk of cancer; ‘Well worth the visit to minimise risk of cancer spreading.’
5. Staff factors (*n* = 31; 15.9%), including knowing that the staff member performing screening would be female, knowing the person conducting the screening, and having a good experience with the person conducting screening; ‘Would feel less intrusive if the procedure could be conducted by a female.’
6. Perceived responsibilities (*n* = 27; 13.8%) including to their own health to attend for screening and responsibilities to family. ‘A part of what it is to be a woman and the things we have to do in order to protect ourselves’
7. The influence of adverts/media/social media or reminders for screening (*n* = 24; 12.3%). This included hearing other’s stories on social media and being aware of screening due to the death of Jade Goody. ‘Celeb stories like Jade Goody make you realise that the threat is real and it can happen to anyone.’
8. Awareness that screening is recommended and important for their health (*n* = 21; 10.8%). ‘It is recommended by friends, family, health workers, government, etc. to get checked out so obviously important.’
9. Having had a good previous experience of screening (*n* = 18; 9.2%); ‘I’ve had lovely staff perform the procedure in the past that put me well at rest.’
10. Feeling family/friend pressure/ support (*n* = 15; 7.7%), which included feeling either pressurised by family to attend screening, or otherwise being supported by family/friends to attend; ‘If you have your family and friends support then you will be more likely to attend a smear.’

Comments that did not fit into any of these broad categories included the need to change the screening method (*n* = 6; 3.1%), knowing statistics on cancer (*n* = 3; 1.5%), and perceiving screening as painless (*n* = 2; 1.0%).

#### Subgroup comparisons

No significant subgroup differences were found for the facilitators reported. In all subgroups of women, the most commonly reported facilitator was the ease of appointment, followed by fear of cancer/to prevent serious illness, and peace of mind.

### Rating of barriers and facilitators

#### Barrier ratings

Across the 194 participants, fear of pain had the highest average rating (Mean = 6.39, SD = 3.11), followed by embarrassment (Mean = 6.18, SD = 3.06), putting screening off (Mean = 5.87, SD = 2.97), having difficulties with making the appointment (Mean = 5.45, SD = 2.98), and competing demands for time (Mean = 5.22, SD = 3.10). The barrier with the average lowest rating was lack of support from family/friends (Mean = 2.80, SD = 2.47; Table 2).

**Table 2.** Descriptive statistics (Means and standard deviations) for barrier and facilitator ratings.

Barrier	Mean	SD	Facilitator	Mean	SD
Fear of pain	6.39	3.12	Belief screening is potentially life-saving	9.03	1.65
Embarrassment	6.18	3.06	Reassurance of finding out everything is OK	8.61	1.83
Putting it off	5.87	2.97	Responsibility for keeping myself healthy	7.63	2.36
Difficulty making appointment	5.45	2.98	Recommended for women my age	7.16	2.51
Competing demands for time	5.23	3.10	Knowing someone with cancer	6.73	2.95
Low perceived risk	4.90	2.97	I can make an appointment at a time that suits me	6.71	2.98
Negative previous experience	4.85	3.25	Positive previous experience	6.39	3.08
Not necessary for women like me	3.09	2.64	Routine	6.16	3.00
Additional costs	3.06	2.51	Media support for screening	5.99	2.98
Lack of support from family/friends	2.80	2.47	Friends and family support screening	5.11	3.20

#### Subgroup comparisons

Multivariate analysis of variance was conducted to assess the interactions between age, deprivation, and previous screening behaviour on the barrier ratings. Entering the ratings of the barriers as outcome measures, there were no interactions found between any of the three subgroups (*ps* > .06).

#### Facilitator ratings

The facilitators had a higher average rating compared to the barriers. Belief screening is potentially life-saving (Mean = 9.03, SD = 1.65) had the highest average rating, followed by the reassurance of finding out everything is OK (Mean = 8.61, SD = 1.82), perceiving screening as part of their responsibility for keeping themselves healthy (Mean = 7.62, SD = 2.36), screening is recommended for women my age (Mean = 7.16, SD = 2.51) and knowing someone with/who has had cancer (Mean = 6.73, SD = 2.95). The facilitator with the lowest average rating was having family or friends that support me to go for screening (Mean = 5.11, SD = 3.20).

#### Subgroup comparisons

A significant three-way interaction was found between deprivation × age × previous screening behaviour for the rating of Belief screening is potentially lifesaving, *F*(4, 125) = 3.83, *p *= .006. Older, overdue/never screened women from the least deprived areas reported this as significantly less important.

Significant two-way interactions were also found between age × deprivation for the belief screening is potentially lifesaving, *F*(4, 125) = 3.04, *p* = .02 and responsibility for keeping myself healthy, *F*(4, 125) = 2.97, *p* = .02. An interaction was also found between age × previous screening behaviour, *F*(1, 137) = 6.39, *p* = .01, for the rating of Responsibility for keeping myself healthy.

A significant main effect of previous screening behaviour was found for the ratings of Belief screening is potentially life-saving, *F*(1, 137) = 20.39, *p* < .001, Responsibility for keeping myself healthy, *F*(1, 137) = 13.52, *p* < .001; I can make an appointment at a time that suits me, *F*(1, 137) = 4.24, *p* = .04. Women who were up-to-date with their screening rated two of these facilitators significantly higher than the overdue/never screened women: Belief screening is potentially life-saving (*Up-to-date*: Mean = 9.46, SD = 1.08 vs. *overdue/never screened*: Mean = 8.20, SD = 2.17, *p* < .001), Responsibility for keeping myself healthy (*Up-to-date*: Mean = 8.22, SD = 1.82 vs. *overdue/never screened*: Mean = 6.47, SD = 2.82, *p* < .001). Overdue/never screened women rated I can make an appointment at a time that suits me as significantly more important for their decision making than the up-to-date women (*Up-to-date*: Mean = 6.48, SD = 3.08 vs. *overdue/never screened*: Mean = 7.14, SD = 2.74, *p* = .04; Table 3).

**Table 3.** Mean ratings and standard deviation (SD) comparison between up-to-date and overdue/never screened women.

	Descriptive statistics				
			Mean	SD	*p*
Barriers	Fear of pain	Up-to-date	6.34	3.06	0.7
Barriers		Overdue/Never screened	6.48	3.24	
	Embarrassment	Up-to-date	5.89	3.19	0.13
		Overdue/Never screened	6.73	2.71	
	Putting it off	Up-to-date	5.68	3.06	0.09
		Overdue/Never screened	6.24	2.78	
	Competing demands for time	Up-to-date	4.82	3.05	0.03
		Overdue/Never screened	6.01	3.06	
	Difficult making appointment	Up-to-date	5.59	3	0.86
		Overdue/Never screened	5.2	2.94	
	Low perceived risk	Up-to-date	4.6	2.88	0.38
		Overdue/Never screened	5.47	3.07	
	Negative previous experience	Up-to-date	5.09	3.2	0.25
		Overdue/Never screened	4.36	3.31	
	Not necessary	Up-to-date	2.73	2.5	0.24
		Overdue/Never screened	3.77	2.77	
	Additional costs	Up-to-date	3.05	2.54	0.98
		Overdue/Never screened	3.07	2.44	
	Lack of support from family/friends	Up-to-date	2.79	2.51	0.86
		Overdue/Never screened	2.83	2.39	
Facilitators	Belief screening is potentially life-saving	Up-to-date	9.46	1.08	<.001
Facilitators		Overdue/Never screened	8.2	2.17	
	Reassurance of finding out everything is OK	Up-to-date	8.86	1.6	0.24
		Overdue/Never screened	8.12	2.13	
	Responsibility for keeping myself healthy	Up-to-date	8.22	1.82	<.001
		Overdue/Never screened	6.47	2.82	
	Recommended for women my age	Up-to-date	7.56	2.35	0.07
		Overdue/Never screened	6.37	2.63	
	I can make an appointment at a time that suits me	Up-to-date	6.48	3.08	0.04
		Overdue/Never screened	7.14	2.74	
	Knowing someone with/who has had cancer	Up-to-date	6.83	2.95	0.77
		Overdue/Never screened	6.54	2.96	
	Positive previous experience	Up-to-date	6.47	3.04	0.99
		Overdue/Never screened	6.22	3.17	
	Media support for screening	Up-to-date	6.19	2.96	0.77
		Overdue/Never screened	5.6	2.98	
	Screening is routine for me	Up-to-date	6.76	2.92	0.1
		Overdue/Never screened	4.98	2.81	
	Friends and family support screening	Up-to-date	4.97	3.35	0.37
		Overdue/Never screened	5.38	2.87	

Note: Up-to-date *N* = 128, Overdue/never screened *N* = 66; *p* values are the result of multivariable ANOVA comparisons with previous screening experience as the between subjects factor.

## Discussion

The present investigated both barriers and facilitators to cervical cancer screening attendance experienced by women living in the UK, along with asking women to explain why these influenced their decision-making. The study included women aged between 25 and 64 and was not limited by levels of socioeconomic deprivation. The online survey design had the double benefit of recruiting a large number of women to provide qualitative responses and reducing some of the barriers to taking part in research, particularly among working-age women. It also included both women up-to-date with their screening and those overdue/never screened which allowed comparison between these subgroups of women. A total of 41 barriers and 46 facilitators were identified via free-text comments. Time, pain/discomfort, and embarrassment were the most commonly reported barriers, and the ease of making an appointment, peace of mind, and fear of cancer/screening being potentially lifesaving were the most commonly reported facilitators.

It is perhaps not surprising that one of the primary barriers to screening was lack of time, considering the majority of individuals invited for screening are of working age and therefore have to juggle work and attending screening. This is a particular issue in the UK where screening is primarily conducted in general practice surgeries which tend to only be open from 9am to 5pm and therefore attending screening requires taking time off from work for the majority of women. A large number of women also reported difficulties with managing childcare in order to attend screening.

The screening was described as ‘exposing’ and ‘invasive’ and participants also reported both pain and the fear of pain as barriers to attending screening. This suggests that negative experiences of screening can influence both the individual and others by sharing their negative experiences. A minority of women also experienced additional issues which make screening attendance difficult, including irregular periods and trauma from past sexual assault. However, sharing positive stories of others that had attended screening and had issues caught early along with information about what the screening involved were also identified as a potential way to encourage attendance.

Many of the facilitators reported were more hypothetical in nature and included suggestions of how the screening experience could be improved to encourage uptake. These tended to focus on making screening easier, both to make appointments for screening (online or via text rather than telephoning a GP surgery) and to attending screening. Participants suggested that the appointments could be conducted in purpose-made vans located in town centres and car parks, to enable screening during lunchtimes or before/after work. It was also suggested that specialist clinics could be set up to deal with a number of issues at one time.

As woman described screening is ‘a part of what it is to be a woman and the things we have to do to protect ourselves’. The life-saving and potentially protective nature of attending screening was shared by just under a quarter of participants. Screening was considered to be a part of their own responsibility for both themselves and their family. It was also considered to be a good way to alleviate worry and provide ‘peace of mind’. This was not just worry on the part of the participant, they also reported that they attended screening to reduce worry for their partners and other family members. Many participants also considered screening to be routine and it was described as similar to going to the dentists or donating blood.

The average ratings of the facilitators were higher than the barrier ratings, suggesting there was greater consensus among the participants in regard to the importance of the facilitators to decision-making. There were also significant differences between the ratings given by women who were up-to-date with screening compared with those that were overdue or never screened. The never screened/overdue women rated perceiving screening being potentially lifesaving and a part of their own responsibility to keep themselves healthy as significantly less important facilitators compared to the up-to-date women. These women also reported the ability to make an appointment at a time that suited them as significantly more important to their decision making, compared to up-to-date women. Little previous work has focused on the facilitators to screening, yet the present study has identified a number of potential differences in the perceptions of these facilitators.

The results of the free-text analysis are broadly consistent with previous research into the barriers to screening which has demonstrated that lack of knowledge, embarrassment, fear of pain, and logistical factors were the most commonly reported (Bukowska-Durawa & Luszczynska, 2014; Ferdous et al., 2018; Hope et al., 2017; Leach & Schoenberg, 2007; Ramjan et al., 2016). These results demonstrate the range of potential barriers encountered by both women that attend for screening and those that put it off or have never attended. No differences were found in the specific barriers reported, or the ratings of the importance of different barriers among the previously screened versus never screened women.

One of the key findings the present study identifies is that while experiencing barriers to attend screening are common and are experienced by both up-to-date and overdue/never screened women, the experience of different facilitators to screening may be more influential over screening attendance. Further research is needed to investigate the influence of these facilitators, how they interplay with the barriers to screening, along with whether interventions may be able to harness these to increase informed screening uptake.

Consistent with the literature, we found that a lower proportion of women from the two quintiles of greater deprivation were currently up to date with screening (66.2% vs. 76.4% least deprived two quintiles). Despite this, we did not find any difference in the barriers or facilitators reported in women from more or less deprived areas. This finding is surprising, and difficult to reconcile. One possible explanation for this could be that women tend to experience the same barriers, regardless of their level of deprivation, however, it remains the women from more deprived areas whose behaviour is more impacted by these barriers. Further detailed investigation of these issues is required.

The barriers and facilitators identified could also be used to improve the screening experience of women by targeting the key factors identified, including highlighting the potential life-saving influence of screening and emphasising regular screening as a method of keeping oneself healthy. The development of public health interventions might additionally consider focusing on these potential factors to increase informed uptake of cervical cancer screening.

Reviews of interventions to improve cervical screening uptake have supported the effectiveness of invitations and reminders, (Duffy, Myles, Maroni, & Mohammad, 2016), which are routine components of the NHS cervical screening programme. This might explain why the UK reports one of the highest rates of screening uptake in Europe. Recent studies also support improving ease of making appointments by enabling online appointment booking (Ryan, Waller, & Marlow, 2019), along with changing methods of screening (Pike, 2019; Yeh, Kennedy, de Vuyst, & Narasimhan, 2019) to increase uptake. The present study also supports the need for practical changes in order to make it as easy as possible for women to attend screening. Suggestions from the individuals who participated in our study included making screening available in locations easy to get to during work lunchbreaks or before/after work, along with clinics which could deal with a number of issues at one time. The present study would support the need for additional focus on exploring the beliefs of overdue/never screened women, particularly the perception that screening is lifesaving and part of one’s own responsibility to keep oneself healthy.

Limitations of the study include the use of two separate scales to assess the importance of the barriers and facilitators meaning the importance of these cannot be directly compared. The sample recruited was limited to mainly White British women and therefore does not represent the experiences of Black, Asian and minority ethnic (BAME) women. This represents the breakdown of ethnic backgrounds for women in the UK who are active on the recruitment site used (Prolific.ac), 90% of women aged 25–64 report their ethnicity as White, with around 10% of women from other ethnic backgrounds. While the most recent census data from the UK (UK Government, 2011) does not break down ethnicity by age and gender, the overall ethnic breakdown of the UK as a whole supports around 80% of the population as White. The present sample may therefore under-represent the views of non-White women. This is a limitation as previous research suggests that cervical screening attendance is particularly low in these women.

Additionally, the subgroups of participants are small and these comparisons are likely to be underpowered. However, the primary focus of the study was to qualitatively identify the perceived barriers and facilitators to screening attendance. There are also some limitations to using this online survey method. For example, participants may not have gone into as much depth with their responses as they might if a face-to face qualitative interview method was used. As part of our work in this area, we have also conducted purely qualitative work (the results of this are not yet published). This work included face to face and telephone interviews to discuss the barriers and facilitators to screening. However, one issue we experienced when recruiting for this interview study was selection bias. Despite recruiting via social media as well as through links within women’s health groups and community groups, the participants we were able to recruit tended to be women who had previously attended screening as well as those who had a generally positive outlook on cervical cancer screening. Participants also tended to be those that were of a higher socio-economic status who were willing to be involved in University research and who were able to take the time to be interviewed.

The present online study aimed to overcome a number of these barriers. Participants were recruited from an online study recruitment website to an online survey that did not take a great deal of time to complete. The recruitment website offers participation in a range of academic and other research in exchange for payment. We were also able to screen participants initially by their socio-economic status and whether or not they regularly attended screening. This provided us with responses from women with a range of experiences in regard to screening. The study included individuals from areas of the greatest deprivation and those who are overdue for screening/have never been screened. Together these two groups of women are considered hard-to-reach and tend to be underrepresented in research studies.

This study provides evidence emphasising the influence of facilitators to cervical cancer screening attendance. Despite the UK reporting one of the highest uptake rates for screening in Europe, women suggested several improvements that might make attending easier and improve uptake. These included making appointments via text or online and screening being carried out in mobile screening vans or clinics which could be accessed easily around work hours and childcare arrangements, along with clinics that deal with a number of issues at once. Psychological facilitators included the peace of mind that screening brings and the belief that cervical cancer screening is potentially life-saving. Barriers to attendance including pain and fear of pain, embarrassment and body issues were identified regardless of their age, socioeconomic deprivation or previous screening behaviour. These findings suggest that most women experience barriers yet continue to attend screening. Public health interventions may therefore usefully focus on the factors which facilitate screening and how these interplay with barriers in order to improve uptake.

## Supplementary Material

Supplemental Material

## Data Availability

Data are available from the corresponding author by request.

## References

[CIT0001] Blomberg, K, Tishelman, C, Ternestedt, B-M, Törnberg, S, Levál, A, & Widmark, C. (2010). How can young women be encouraged to attend cervical cancer screening? Suggestions from face-to-face and internet focus group discussions with 30-year-old women in Stockholm, Sweden. *Acta Oncologica*, *50*(1), 112–120. doi:10.3109/0284186X.2010.528790. 21091087

[CIT0002] Bukowska-Durawa, A., & Luszczynska, A. (2014). Cervical cancer screening and psychosocial barriers perceived by patients. A systematic review. *Contemporary Oncology*, *18*(3), 153–159. doi: 10.5114/wo.2014.43158 25520573 PMC4269002

[CIT0003] Cancer Research UK. (2015). *Cervical cancer statistics*. Retrieved from https://www.cancerresearchuk.org/health-professional/cancer-statistics/statistics-by-cancer-type/cervical-cancer#heading-Zero

[CIT0004] Cervical Screening Wales. (2019). Cervical Screening Wales Annual Screening Statistics Report. Retrieved from http://www.cervicalscreeningwales.wales.nhs.uk/sitesplus/documents/1032/Cervical%20Screening%20Wales%20Annual%20Statistical%20Report%202018-19.V1.0.pdf.

[CIT0005] Chorley, A. J., Marlow, L. A. V., Forster, A. S., Haddrell, J. B., & Waller, J. (2017). Experiences of cervical screening and barriers to participation in the context of an organised programme: A systematic review and thematic synthesis. *Psycho-Oncology*, *26*(2), 161–172. doi: 10.1002/pon.4126 27072589 PMC5324630

[CIT0006] Douglas, E., Waller, J., Duffy, S. W., & Wardle, J. (2016). Socioeconomic inequalities in breast and cervical screening coverage in England: Are we closing the gap? *Journal of Medical Screening*, *23*(2), 98–103. doi: 10.1177/0969141315600192 26377810 PMC4855247

[CIT0007] Duffy, S. W., Myles, J. P., Maroni, R., & Mohammad, A. (2016). Rapid review of evaluation of interventions to improve participation in cancer screening services. *Journal of Medical Screening*, *24*(3), 127–145. doi: 10.1177/0969141316664757 27754937 PMC5542134

[CIT0008] Elo, S., & Kyngäs, H. (2008). The qualitative content analysis process. Journal of advanced nursing. *Journal of advanced nursing*, *62*(1), 107–115. doi: 10.1111/j.1365-2648.2007.04569.x 18352969

[CIT0009] European Council. (2003). Council recommendation on cancer screening. Retrieved from https://screening.iarc.fr/doc/cancer_screening.pdf.

[CIT0010] Ferdous, M., Lee, S., Goopy, S., Yang, H., Rumana, N., Abedin, T., & Turin, T. C. (2018). Barriers to cervical cancer screening faced by immigrant women in Canada: A systematic scoping review. *BMC Women’s Health*, *18*(1), 165. doi: 10.1186/s12905-018-0654-5 30305056 PMC6180489

[CIT0011] Gianino, M. M., Lenzi, J., Bonaudo, M., Fantini, M. P., Siliquini, R., Ricciardi, W., & Damiani, G. (2018). Organized screening programmes for breast and cervical cancer in 17 EU countries: Trajectories of attendance rates. *BMC Public Health*, *18*(1), 1236. doi: 10.1186/s12889-018-6155-5 30400786 PMC6220470

[CIT0012] Hope, K. A., Moss, E., Redman, C. W. E., & Sherman, S. M. (2017). Psycho-social influences upon older women’s decision to attend cervical screening: A review of current evidence. *Preventative Medicine*, *101*, 60–66. doi: 10.1016/j.ypmed.2017.05.002 28502577

[CIT0013] HSC Public Health Agency. (2018). Northern Ireland Cervical Screening Programme Factsheet. Retrieved from http://www.cancerscreening.hscni.net/pdf/FACTSHEET-2017-2018%20%20%203-5y%20coverage.pdf.

[CIT0014] Leach, C. R., & Schoenberg, N. E. (2007). The vicious cycle of inadequate early detection: A complementary study on barriers to cervical cancer screening among middle-aged and older women. *Preventing Chronic Disease*, *4*(4), A95. 17875270 PMC2099293

[CIT0015] NHS Digital. (2019). *Cervical screening programme England, 2018-19*. https://www.gov.uk/government/statistics/cervical-screening-programme-england-2018-19

[CIT0016] O’Connor, M., Murphy, J., Martin, C., O’Leary, J., & Sharp, L. (2014). Motivators for women to attend cervical screening: The influential role of GPs. *Family Practice*, *31*(4), 475–482. doi: 10.1093/fampra/cmu029 24927724

[CIT0017] Peirson, L., Fitzpatrick-Lewis, D., Ciliska, D., & Warren, R. (2013). Screening for cervical cancer: A systematic review and meta-analysis. *Systematic Reviews*, *2*(1), 35. doi: 10.1186/2046-4053-2-35 23706117 PMC3681632

[CIT0018] Pike, H. (2019). HPV self testing to be piloted in two areas. *BMJ*, *364*, l1357. doi:10.1136/bmj.l1357%J doi: 10.1136/bmj.l1357%J 30910781

[CIT0019] Public Health Scotland. (2020). Cervical Cancer Screening. Retrieved from https://www.isdscotland.org/Health-Topics/Cancer/Cervical-Screening/.

[CIT0020] Ramjan, L., Cotton, A., Algoso, M., & Peters, K. (2016). Barriers to breast and cervical cancer screening for women with physical disability: A review. *Women & Health*, *56*(2), 141–156. doi: 10.1080/03630242.2015.1086463 26325597

[CIT0021] Ryan, M., Waller, J., & Marlow, L. A. (2019). Could changing invitation and booking processes help women translate their cervical screening intentions into action? A population-based survey of women’s preferences in Great Britain. *BMJ Open*, *9*(7), e028134. doi:10.1136/bmjopen-2018-028134%J doi: 10.1136/bmjopen-2018-028134%J PMC6629419 31300499

[CIT0022] UK Government. (2011). Ethnicity Facts and Figures. Retrieved from https://www.ethnicity-facts-figures.service.gov.uk/uk-population-by-ethnicity.

[CIT0023] World Health Organisation. (2018). *Early diagnosis and screening: Cervical cancer*. Retrieved from https://www.who.int/cancer/prevention/diagnosis-screening/cervical-cancer/en/

[CIT0024] Yeh, P. T., Kennedy, C. E., de Vuyst, H., & Narasimhan, M. (2019). Self-sampling for human papillomavirus (HPV) testing: A systematic review and meta-analysis. *BMJ Global Health*, *4*(3), e001351. doi:10.1136/bmjgh-2018-001351%J doi: 10.1136/bmjgh-2018-001351%J PMC6529022 31179035

