# Supplementary material for: What factors are most influential in increasing cervical cancer screening attendance? An online study of UK-based women
Source: Health Psychol Behav Med. 2020 Aug 7;8(1):314–28. doi: 10.1080/21642850.2020.1798239 (PMC8114340; doi:10.1080/21642850.2020.1798239)
Supplement: Supplemental Material [file RHPB_A_1798239_SM4179.docx]

## Supplementary files

**File 1. Online survey.**

**Opinions on cervical cancer screening**

1. Please enter your Prolific Academic ID
2. What is your age?
3. What is your nationality?
4. How would you best describe your ethnicity?

- Caucasian
- Mixed
- African
- Latino/Hispanic
- East Asian
- South Asian
- Middle Eastern
- Caribbean
- Other

1. Please enter your postcode - this is to give us a better idea of where people responding to the survey are located.
2. Are you currently registered with a UK GP?

- Yes
- No

1. If you have been invited to attend cervical cancer screening: When was the last time you attended cervical cancer screening?

- Within the last year
- Within the last 1-3 years
- Within the last 3-5 years
- More than 5 years ago
- Not applicable
- I would rather not say

**Barriers to screening**

1. What are the sorts of things that might put you off going for screening? – Please list all the different things you can think of and briefly explain why.

Reason 1

Why might it put you off going for screening?

Reason 2

Why might it put you off going for screening?

Reason 3

Why might it put you off going for screening?

Reason 4

Why might it put you off going for screening?

Reason 5

Why might it put you off going for screening?

1. Can you rate this list of **barriers** that might stop you going for cervical cancer screening by which you think would be **least**to **most**important for your decision making?

|  | 1 Least important | 2 | 3 | 4 | 5 | 6 | 7 | 8 | 9 | 10 Most important |
| --- | --- | --- | --- | --- | --- | --- | --- | --- | --- | --- |
| Embarrassment |  |  |  |  |  |  |  |  |  |  |
| Fear of pain |  |  |  |  |  |  |  |  |  |  |
| Putting it off/not getting around to making appointment |  |  |  |  |  |  |  |  |  |  |
| Low perceived risk of cervical cancer |  |  |  |  |  |  |  |  |  |  |
| Difficulty making appointment |  |  |  |  |  |  |  |  |  |  |
| Additional costs associated with attending screening |  |  |  |  |  |  |  |  |  |  |
| It is not necessary for women like me |  |  |  |  |  |  |  |  |  |  |
| Negative previous experience |  |  |  |  |  |  |  |  |  |  |
| Competing demands for my time/other things are more important |  |  |  |  |  |  |  |  |  |  |
| Lack of support from family/friends |  |  |  |  |  |  |  |  |  |  |
|  |  |  |  |  |  |  |  |  |  |  |

1. Are there any barriers not mentioned above that you think should have been listed in the previous question? – if yes, where would you rank this?

**Things that encourage me to go for screening**

1. What are the things that would/do encourage you to go for screening? – Please list them and briefly explain why

Reason 1

Why might it encourage you to go for screening?

Reason 2

Why might it encourage you to go for screening?

Reason 3

Why might it encourage you to go for screening?

Reason 4

Why might it encourage you to go for screening?

Reason 5

Why might it encourage you to go for screening?

1. Can you rate this list of reasons that might **encourage** you to go for cervical cancer screening by which you think would be **least**to **most**important for your decision making?

|  | 1 Least important | 2 | 3 | 4 | 5 | 6 | 7 | 8 | 9 | 10 Most important |
| --- | --- | --- | --- | --- | --- | --- | --- | --- | --- | --- |
| Belief that screening is potentially life-saving |  |  |  |  |  |  |  |  |  |  |
| Reassurance of finding out everything is OK |  |  |  |  |  |  |  |  |  |  |
| My friends and family support me to go for screening |  |  |  |  |  |  |  |  |  |  |
| It is part of my responsibility of keeping myself healthy |  |  |  |  |  |  |  |  |  |  |
| It is recommended for women my age |  |  |  |  |  |  |  |  |  |  |
| Knowing someone with/who has had cancer |  |  |  |  |  |  |  |  |  |  |
| Information in the media supports screening |  |  |  |  |  |  |  |  |  |  |
| Screening is routine for me |  |  |  |  |  |  |  |  |  |  |
| I can make an appointment at a time that suits me |  |  |  |  |  |  |  |  |  |  |
| Positive previous experience of screening |  |  |  |  |  |  |  |  |  |  |

1. Are there any other reasons not mentioned that you think should be listed? Where would you rate them on the scale above?

## Supplementary Table 1. List of barrier themes, the number of comments reporting these and quotes representing each in the overall sample.

|  | **Barrier codes** |  |
| --- | --- | --- |
| Time (N = 95) | Time | "Hard to fit in an appt that isn't urgent"; "It is hard to find time during my regular working hours to schedule a screening, as I work 7 days a week."; "No time to go to the doctors" |
|  | Other commitments | "Fitting the apointment around school times and childcare for the younger 2 children"; "Fitting the appointment around childcare, work, etc."; "I work full time and doctors isn’t open at weekends"; "never find time" |
|  | Busy | "busy lifestyle, unable to get time off work" |
|  | Forgetting | "its easy to forget to make the appointment and not be reminded."; "Time slips away and you think it was more recent than it was" |
| Pain/discomfort/fear of pain (N = 131) | Discomfort | "I don't like the discomfort it causes"; "Wanting to avoid any pain or discomfort"; "Discomfort of the procedure"; "I think the experience will be uncomfortable" |
|  | Pain | "I dont want to go through pain"; "Sometimes it can be painful"; "I find the examination very painful so it's something I dread" |
|  | Fear of pain | "It can be quite painful so it may be a scary experience for some people."; "Because it might hurt"; |
| Appointment availability/difficulty (N = 68) | Difficulty with appointment | "My GP surgery operated between 8 and 5 weekdays only, it is very difficult to get an appointment when I work full time. It is made even more difficult as the nurses are not available everyday, only on certain days during the week.; "I would be more likely to go if I was able to get an appointment either before or after work"; "It can be difficult to fit the appointment into a busy schedule" |
|  | Childcare | "i have 2 young toddlers so childcare is needed"; "fitting the apointment around school times and childcare for the younger 2 children"; "Its not the kind of thing that i can take my daughter too with me"; "not wanting to take children along" |
| Embarrassment (N = 113) | Embarrassment | "embarrassed to bare all to nurses"; "find the whole process embarrassing" |
|  | Anxiety | "Scared you will be judged and have anxiety about it"; "I suffer with this anyway so it effects me going getting cervical testing done."; "I have anxiety and depression" |
|  | Nervous | "Nervous about it"; "I hate the wait for results it makes me nervous"; "Nervous and embarrassing" |
|  | Shy | "Being shy"; "I am a bit shy of my body"; "FEELING SHY ABOUT HAVING THE PROCEDURE" |
|  | Invasive | "The thought of having someone scraping cervix is uncomfortable"; "I would feel violated"; "Scary to have an invasive process when it is unknown what it will be like" |
|  | Self-conscious | "Just self consciousness"; "Feeling self conscious at the intimacy involved." |
| Body issues (N = 35) | Body issues | "Purely embarrassment over appearance."; "Not wanting to have to take clothes off in front of people (even if they are medical professionals who've "seen it all before") due to body issues."; "Fear of a smell" |
|  | Period | "irregular periods because of implant"; "i did put off screening for 2 years after i was invited as i was on the implant and bled continuously so didn't really know when my period was"; "Completely erratic and unregular periods which mean I can bleed for months on end nearly put me off going for a smear as the appointment had to be rearranged about six times and the receptionist was getting irritated by it." |
| Fear of results (N = 59) | Fear of results | "Because I would be worried incase the results were abnormal"; "Not wanting bad results"; "I hate the wait for results it makes me nervous"; "fear of finding out something is actually wrong." |
|  | Fear | “If you're afraid of something sometimes you can't force yourself to go.” |
| Low perceived risk (N = 30) | Low risk | sometimes you can't imagine you're at risk and you don't get round to it; "I almost certainly do not have HPV due to my limited sexual contact. My husband and I have only ever had sexual contact with each other." |
|  | Not important | “Apathy”; “Not thinking it's important”; “Because you think its not important” |
|  | No symptoms | “Feeling healthy, so assuming that you don't need a smear test.” |
| Previous experience (N = 18) | Previous experience | I have previously had a very poor experience of care with gynecology; "Bad experience of nurse care"; "very first screening was an uncomfortable experience that left me in discomfort for several days afterward." |
| Nervous/unsure of process (N = 20) | Unsure of process | Fear of the unknown for first timers; "Scary to have an invasive process when it is unknown what it will be like"; "Because you don't know what to expect"; "I am unsure of what the procedure entails" |
| GP/Staff factors (N = 16) | GP issues | "After the last time i went to the doctors with 'women's issues' it took them 25 minutes of prodding about to find a lump almost plum sized- they tried to tell me it wasn't there. Then I had surgery to fix this- months of side effects including pot-operative shock and constipation (not fun with vaginal stitches!). I have no trust with my village doctors,"; "unfriendly surrounding" |
|  | male nurse | "not know if the nurse will be a female or a male"; "I wouldn't want a male to do it." |
|  | Staff issues | "When I have attended other appointments the nurse or practitioner has not been warm or friendly to encounter. This always puts me going to the doctor regardless of the issue."; "You may worry that the nurse isn't competent at carrying out the test." |
|  | Nurse issues | "Not thinking the nurse is particually friendly"; "I have previously had a very poor experience of care with gynecology" |
|  | Knowing Nurse | "Knowing the nurse would be uncomfortable" |
|  | Judgement from nurse | "The thought of not looking normal down below"; "In the past I have been "told off" by health professionals for things like choosing to shave my pubic hair or for objecting to having a male doctor present without being informed beforehand. I was made to feel like I was making trouble and was called discriminatory" |
|  | Unfriendly staff | “Worried staff may not put you at ease” |
|  | Horror stories | "You hear so many things about people getting wrong results"; "Hearing stories from friends about their own experiences" |
|  | Past sexual assault | "Last nurse persisted with using a speculum which was agony for about 15 minutes (despite being a SSA victim). She then did the smear without me even realising by using a longer speculum and tried to cover it up so I wouldn't realise. You expect some discomfort but this was actual pain."; |
|  | Childbirth | "Bad birthing experience"; "After having a lot of stitches after child birth it's hard to relax and feel comfortable" |
|  | Dislike being touched | "Was raped in the past, find it difficult to allow anyone to look/touch my intimate parts"; "I don't like to be touched, I have sensory issues, I struggle socially, with noises and bright lights." |
|  | Accessibility issues | "Those who are disabled may not be able to have a smear because their practise doesn't cater for their needs." |
|  | Denial | "If there were any abnormalities detected, then it would be a serious concern that would have to be dealt with. It might just be easier to be unaware of any potential implications." |
|  | High risk | “High level of risk” |

## Supplementary Table 2. List of facilitator themes, the number of women reporting these and quotes representing each in the overall sample.

| **Broad category** | **Codes** | **Quotes** |
| --- | --- | --- |
| Ease of appointment (N = 71) | Ease of appointment | "If I could make same day appointments at a time to suit me around my children /childcare arrangements."; "Being able to attend a clinic that does not interfere with my other commitments."; "Later appointments" "Easily being able to make an appointment"; "if it was easy to get an appointment and attend" |
|  | Location | Option of clinic rather than just doctors; "Rather than booking an appointment (which are of limited availability) it would be easier to have drop in clinics." |
|  | Childcare | “To fit around work/childcare”; “Somewhere for children to wait rather than being in room” |
| Peace of mind (N = 48) | Peace of mind | Peace of mind; "Just to be on the safe side"; "Reassurance" |
|  | Reassurance | "It would rest my mind to know i was ok after a screening"; "to be reassured you are ok"; "I would know for sure where I stand" |
|  | Finding out if there are problems | "Finding out if there are problems" |
|  | Identifying issues | "PICKING UP ANY EARLY WARNING SIGNS" |
| Good past experience (N = 18) | Previous experience | "I've had lovely staff perform the procedure in the past that put me well at rest"; "Good experiences of other women tell me that it might not be too bad"; "if you go the first time and have a positive experience then this would encourage me to return in the future" |
| Fear of cancer/Prevent serious illness (N = 48) | Potentially life saving | That screening may save me from it; "It's a free, potentially life saving, simple check-up. Why would I not have it done?"; |
|  | Fear of cancer | "Because it is the only way of finding out"; "Scared of cancer" |
|  | Prevent serious illness | Checking my smear could catch early signs of cancer and make treatment less invasive; "To try and find out as early and possibly to have a higher chance of treating it"; "want to stop any risks of getting cancer" |
|  | Early enough to treat | "not wanting to get cancer and if it happens getting it caught early; "Catching it early is important."; "Should anything be a miss it’s better to catch things as soon as possible" |
| Perceived risk of cancer (N = 38) | Risk of cancer | Well worth the visit to minimise risk of cancer spreading; "I wouldn't want to risk anything happening to me that would hurt my family."; "Risk of cancer in family" |
|  | Family history | "If a close family member had the cancer it would then encourage me to go and get checked out"; "seeing people I know experience cancer motivates me to attend screening" |
|  | Minimise risk |  |
|  | Past health scares | "previous health scares make it good to know what is going on with your body" |
|  | Knowing someone with cancer | We very nearly lost a family member to cervical cancer recently because she didn't go for her smears through embarrassment. |
| Important (N = 20) | Important | Your health isn't something to second guess; "Catching it early is important."; "I have read information about how important it is to find cervical cancer early and how it can be completely treated if found early. That would encourage me to attend screening." |
|  | Important for health | I want to be 100% healthy as possible; "Its important for my health"; "Its good to know that my health is okay"; "Getting a regular checkup helps you to know that that part of your body is healthy." |
| Family/friend pressure/support (N = 15) | Support from others | More support, expected to go, no excuse ; "If you have your family and friends support then you will be more likely to attend a smear."; "Sometimes your family motivates you to go and see your GP." |
|  | Pressure from others | "If they are doing it, I should do it too"; "friends and family encourage me to go for screening"; "A friends word: It’s always easier when someone you know has had it done"; "TALKING TO FRIENDS THAT HAVE ALREADY HAD THE EXPERIENCE"; |
| Responsibilities (N = 27) | Family responsibilities | I want to have children in the future; "I wouldn’t want my daughters to be too afraid to get checked" |
|  | Own responsibility | I feel like I should do this to make sure that I am healthy so that nothing bad will happen to me.; "I have some responsibility over my health."; "my responsibility to keep healthy"; "A PART OF WHAT IT IS TO BE A WOMAN AND THE THINGS WE HAVE TO DO IN ORDER TO PROTECT OURSELVES" |
|  | Caring for body |  |
| Staff factors (N = 31) | Female nurse | "Would feel less intrusive if the procedure could be conducted by a female"; "knowing that the nurse will be a female" |
|  | Nice staff | "It goes a long way when the staff are nice and put you at ease." "I've had lovely staff perform the procedure in the past that put me well at rest " |
|  | Understanding from staff |  |
| Recommended (N = 21) | Recommended | is recommended to do it; "It's expected people in my age group go."; "is recommended to do it"; "Recommended for age over 30"; "It is recommended by friends, family, health workers, goverment etc to get checked out so obviously important" |
| Adverts/social media/media/reminders (N = 24) | Reminders | "I can forget so reminders help"; "Seeing advertisements encouraging screening, in case it has slipped my mind." |
|  | Adverts | It has helped me to understand why it is important to go for your screening.; "Well-known people talking about it on social media more openly."; "Seeing advertisements encouraging screening, in case it has slipped my mind." |
|  | Social media | Celebrities saying it’s not painful and that they do it breaks the taboo.; "Public endorsement makes it seem like a better idea to go" |
|  | Jade Goody | As she died so young it scared me at the time and now i always make sure i get my smear; "Jade Goody dying of cancer"; "Celeb stories like Jade Goody make you realise that the threat is real and it can happen to anyone" |
|  | Change screening method | "If I didn't have to get undressed and have someone insert things then I'd go"; "Different screening method"; "Doing at home in my own comforts would relax me more." |
|  | Routine | “Once every 18 month to 2 years as opposed to 3, should be more routine as can happen at anytime”; “it being part of your routine, as easy as going to the dentist” |
|  | Knowing statistics | “Knowing death rates and it could happen to anyone”; “Statistics on how many people treated vs tested” |
|  | Painless | “I know that screening isn't painful”; “I had have very nice experiences - quick and swift and painless and also nice staff - making it actually a pleasant social experience.” |
|  | Lucky to have the NHS | “We are lucky to have access to this service.” |
|  | Confidence | “Good records would cheer me up and make me confident.” |
|  | Beneficial for health | “it's beneficial to my health to go” |

## Supplementary Table 3. List of facilitator themes, the number of women reporting these and quotes representing each. Limited to never screened/women overdue for screening.

| **Broad category** | **Codes** |  |  |
| --- | --- | --- | --- |
| Ease of appointment | Ease of appointment | 26 | "if it was easy to get an appointment and attend"; "Easy to just make the appointment online."; "Later appointments"; "Being able to schedule an appointment." |
|  | Location | 2 | “Nearer to work”; “You could use the clinic as a one-stop for female health issues ranging from contraception to gynaecological health in general and thereby save time and numerous appointments.” |
|  | Childcare | 1 | “To fit around work/childcare” |
| Peace of mind | Peace of mind | 8 | "Want to know everything is ok"; "If I worried so much about getting cancer that it persuaded me to go for the screening"; "To ease any worries i may have" |
|  | Reassurance | 4 | “Knowing that I can be given the all clear”; “it would rest my mind to know i was ok after a screening” |
|  | Identifying issues | 1 | “It could let me know if there is some issue” |
| Good past experience | Previous experience | 1 | “if you go the first time and have a positive experience then this would encourage me to return in the future” |
|  | Quick | 6 | “Quickly done as im always busy”; “Won’t be in discomfort for long”; “no time for waiting around” |
| Awareness of process | Being aware of process | 7 | "Fear of the unknown is probably a large factor in women not attending."; "I would know what to expect"; |
|  | More information | 2 | “Having more information and even maybe a video demo to show women if its less painful or not and exactly what to expect, would definitely motivate me more.” |
| Fear of cancer/Prevent serious illness | Potentially life saving | 7 | "life saving procedure"; "nobody wants to be in a position where there is nothing else that can be done for them so going to the screening means that disease may be caught earlier"; |
|  | Fear of cancer | 5 | “If I worried so much about getting cancer that it persuaded me to go for the screening”; “Because it is the only way of finding out” |
|  | Prevent serious illness | 3 | “To get screened could possibly change my life. Early prevention is key to success in fighting cancers.”; “I do not want to suffer” |
|  | Early enough to treat | 2 | “Best to detect early” |
| Perceived risk of cancer | Risk of cancer | 4 | “if i was at risk”; “Understanding the risk of not going.” |
|  | Family history | 2 | “if a close family member had the cancer” |
|  | Past health scares | 1 | “previous health scares make it good to know what is going on with your body” |
|  | Knowing someone with cancer | 2 | “This may make me think that it could happen to me too.” |
| Family/friend pressure/support | Support from others | 6 | “I’d have moral support and someone to “hold my hand””;” It's more supportive than just being alone with a nurse/doctor.” |
|  | Pressure from others | 4 | "if a family member pressured me in to going I would get fed up of it and end up going"; "If members of my family pleaded with me to go I probably would."; "Family encourage" |
| Responsibilities | Own responsibility | 2 | "Feel it is my responsibility" |
| Staff factors | Female nurse | 3 | “Female nurse who I already knew”; “would feel less intrusive if the procedure could be conducted by a female” |
|  | Nice staff | 1 | "would feel less intrusive if the procedure could be conducted by a female"; "Female nurse who I already knew"; "Would feel unsafe with a man" |
|  | Understanding from staff | 1 | “they assume im sexually active so it should be easy im not.understanding im not being difficult and have issues making me feel supported probably wouldnt be so tense” |
| Recommended | Recommended | 4 | “It's expected people in my age group go.” |
| Adverts/social media/media/reminders | Reminders | 2 | “Telling me that I'm due a screening” |
|  | Adverts | 1 | "It makes me feel like I am not alone and other women are concerned about me"; "It keeps reminding you it is important"; " |
|  | Social media | 1 | “It makes me feel like I am not alone and other women are concerned about me” |
|  | Others experiences | 1 | “Good experiences of other women tell me that it might not be too bad” |
|  | Change screening method | 2 | “if it was possible, some people would prefer an at home test you do yourself”; “I would get it done then if it would be not as embarrassing as less invasive.” |
|  | Routine | 2 | “Making screening an automated appointment” |
|  | Beneficial for health | 1 | “it's beneficial to my health to go” |

## Supplementary Table 4. List of barrier themes, the number of women reporting these and quotes representing each. Limited to women never screened before or overdue for screening.

|  | Barrier codes | Count |  |
| --- | --- | --- | --- |
| Time | Time | 24 | “Trying to fit in an appt when always busy"; "Hard to get time off work"; "Trying to fit in a visit around working hours."; "I keep putting it off until the next time." |
|  | Other commitments | 15 | “Hard to get time off work"; "Trying to find the time to fit it in around work can be really hard for me because of my job."; "generally just too busy" |
|  | Busy | 4 | “I work Monday to Friday and am a parent so dont have the time”; “Trying to fit in an appt when always busy” |
| Pain/discomfort/fear of pain | Discomfort | 20 | "I don't like the discomfort it causes"; "I find the whole idea of having a smear deeply uncomfortable" |
|  | Pain | 18 | "I have heard it's uncomfortable so it worries me about how it would feel and if it would be painful"; "Painful procedure? Difficulty obtaining sample, too nervous, embarrassing."; "Heard its painful"; "I have only had female partners and therefore I think it would be very painful/difficult for the test to be carried out. There is never any information specifically related to LGBT patients" |
|  | Fear of pain | 7 | “Heard its painful”; “Pain in that region is quite hard to deal with mentally and I am afraid that it would hurt too much.” |
| Appointment availability/difficulty | Difficulty with appointment | 8 | “time consuming to book appointments with a busy work life for something i don't view as important”; “unable to get an appointment”; “having to keep ringing and not having the time”; “unable to get an appointment outside work hours” |
|  | Childcare | 2 | “i might not want to take my chid with me” |
| Embarassment | Embarrassment | 14 | "Because it is too personal and embarrassing"; "I would find it very embarrassing and would be wondering if I was being judged on how it looks and if I look different to others in that area"; "Really embarrassed to have an intervention like this"; "Not wanting to have to take clothes off in front of people (even if they are medical professionals who've "seen it all before") due to body issues." |
|  | Anxiety | 5 | “Having anxiety would make me even nervous to go and get it done.”; “I have anxiety and depression” |
|  | Invasive | 2 | “I would feel violated” |
|  | Self conscious | 1 | “Feeling self conscious at the intimacy involved.” |
| Body issues | Body issues | 6 | “I have experienced discomfort during previous smears due to medical staff not understanding how to obtain a smear from a patient who's uterus tilts backwards.”; “Not wanting to have to take clothes off in front of people (even if they are medical professionals who've "seen it all before") due to body issues.”; “i dont like showing people bits of my body” |
|  | Period | 3 | ”I have unpredictable periods so making an appointment in advance doesn’t really work as I could get my period an hour before going” |
| Fear of results | Fear of results | 14 | "i would be scared and rather not know"; "Fearing the results"; "Because I would be worried incase the results were abnormal"; "Not wanting bad results" |
|  | Fear | 2 | “I'm afraid there might be something found and I would rather not know” |
| Low perceived risk | Low risk | 12 | "I'm unsure if it is really necessary to have the screening as my chances of having caught HPV are so low?"; "not needed"; |
|  | Not important | 2 | “Laissez faire attitude”; “Does not seem important” |
| Previous experience | Previous experience | 4 | “In the past I have been "told off" by health professionals for things like choosing to shave my pubic hair or for objecting to having a male doctor present without being informed beforehand. I was made to feel like I was making trouble and was called discriminatory”; “At my last screening I suffered quite severe pain from the speculum. Don't want to repeat that” |
| Nervous/unsure of process | Unsure of process | 6 | "I do not know what the procedure requires"; |
| GP/Staff factors | GP issues | 5 | "Unfamiliar staff, unknown attitudes,"; "Unknown staff, no real avenue to ask questions prior aside from wasting a GP's appointment slot"; "judgement from the nurse/gp doing the screening" |
|  | Nurse issues | 3 | "I wouldn't feel comfortable unless it was a female doing it as they are more used to a female body"; "Would prefer a female nurse that I already knew." |
|  | male nurse | 2 | “i would need reassurance that I would have a female nurse”; “if it were a male doctor doing it” |
|  | Staff issues | 2 | “You don’t know who is going to be doing the procedure, it therefore varies from person to person.” “Her attitude. "No need to be embarrassed, I've done 100s of these". Yes but I HAVEN'T” |
|  | Past sexual assault | 2 | “Previous rape and sexual assault mean that it is too triggering to go for a smear, especially when medical staff have been less than sympathetic in the past.” |
|  | High risk | 1 |  |
|  | Dislike being touched | 1 | “I do not want to be touched by strangers. I feel that medical practitioners do not have enough understanding of my condition to make me comfortable.” |
|  | Judgement from nurse | 1 | “Little compassion for personal anxieties or being a first test” |
|  | Horror stories | 1 | “You hear so many things about people getting wrong results” |

Never screened N = 45 women; overdue for screening N = 15.
